# Prevalence and correlates of Alzheimer’s disease and related dementias in rural Uganda: cross-sectional, population-based study

**DOI:** 10.1186/s12877-020-1461-z

**Published:** 2020-02-10

**Authors:** Vincent Mubangizi, Samuel Maling, Celestino Obua, Alexander C. Tsai

**Affiliations:** 1 Mbarara University of Science and Technology, Mbarara, Uganda; 2 Massachusetts General Hospital and Harvard Medical School, Boston, MA USA

**Keywords:** Alzheimer’s disease, Dementia, Sub-Saharan Africa, Uganda

## Abstract

**Background:**

There is a paucity of data on the prevalence and correlates of Alzheimer’s disease and related dementias in sub-Saharan Africa. The aim of the study was to estimate the prevalence and correlates of Alzheimer’s disease and related dementias in rural Uganda.

**Methods:**

We conducted a cross-sectional, population-based study in a rural region of southwestern Uganda. The Brief Community Screening Instrument for Dementia was administered to a multi-stage area probability sample of 400 people aged 60 years and over. Multivariable logistic regression was used to estimate correlates of probable dementia.

**Results:**

Overall, 80 (20%) of the sample screened positive for dementia. On multivariable regression, we estimated the following correlates of probable dementia: age (adjusted odds ratio [AOR], 1.02 per year; 95% confidence interval [CI], 1.10–1.03, *p*<0.001), having some formal education (AOR, 0.57; 95% CI, 0.41–0.81, *p* = 0.001), exercise (AOR, 0.44; 95% CI, 0.27–0.72, *p* = 0.001), and having a ventilated kitchen (AOR, 0.43; (95% CI, 0.24–0.77, *p* = 0.001).

**Conclusions:**

In this population-based sample of older-age adults in rural Uganda, nearly one-fifth screened positive for dementia.

## Background

Alzheimer’s disease, other dementias, and non-communicable diseases are likely to impose an increasing burden on health systems throughout sub-Saharan Africa, as country populations age and as communicable disease mortality and morbidity decline [1, 2]. More than half of all people with dementia are from low- and middle-income countries [1]. A recent report released by Alzheimer’s Disease International estimated the age-adjusted dementia prevalence in Sub-Saharan Africa to be 7.2% among those aged 60 years and over. Globally, a large number of variables have been investigated as potential risk and protective factors for dementia [3], including vascular disease, life style, psychosocial and psychological factors, infectious diseases, genetic factors, and carbon monoxide poisoning.

There are few population-based studies of the prevalence and correlates of dementia in sub-Saharan Africa. The *Dementia in Sub-Saharan Africa: Challenges and Opportunities* report by Alzheimer’s Disease International identified only 12 studies conducted in sub-Saharan Africa; of these, six were conducted in West Africa, and only one study was conducted in East Africa (Tanzania) [2]. Estimating the population prevalence of Alzheimer’s disease and related dementias (AD/RD) in sub-Saharan Africa, and identifying correlates of AD/RD, has been identified as a public health priority [2, 4]. To address this gap in the literature, we conducted a population-based study to estimate the prevalence and correlates of Alzheimer's disease and related dementias (AD/RD) in rural Uganda.

## Methods

### Study design

We conducted a cross-sectional, population-based study in December 2018 in 16 districts of southwestern Uganda. In this region, the local economy is largely dominated by subsistence agriculture, animal husbandry, and petty trading. Food and water insecurity are common [5, 6]. People aged 60 years and older comprise approximately 5% of the total population in the region [7].

Given our study’s focus on AD/RD, we sought to enroll people aged 60 years and older. Study participants were selected using a multi-stage area probability sampling of parishes. In the first stage, using a computerized random number generator, eight districts were selected from a list of 16 districts in southwestern Uganda. In the second stage, one sub-county (or an equivalent administrative local government) was randomly selected from an ordered list of all sub-counties in each of the 8 districts. In the third stage, one parish was randomly selected from each of the selected sub-counties. In each parish, we went door-to-door, recruiting participants consecutively using lists of older-age people that were provided by village health team members, until the required number per parish (50 people) was achieved. Upon approach, potential study participants were asked to confirm their date of birth by furnishing their national identity card. Participants were required to invite a consenting caregiver to answer questions about their activities of daily living. Participants did not need to be literate but were required to hear and see sufficiently well to communicate with study staff, answer survey questions, and undertake cognitive tests.

### Survey instrument

We used the brief Community Screening Instrument for Dementia (CSID),which is suitable for use by non-specialists in low-resource settings [8]. The screening interview has two sections, with one interview for the person suspected of having dementia, which is used to determine the CSID cognitive score (7 items; range, 0–9, higher score being better); and a separate informant section for a caregiver or close relation, which is used to determine the CSID informant score (6 items; range, 0–6, higher score being worse). A CSID cognitive score ≤ 4 suggests probable dementia, a score of 5–6 suggests possible dementia, while a score ≥ 7 suggests dementia is unlikely. For those with a CSID cognitive score of 5 or 6 suggesting possible dementia, we then interviewed the caregiver/informant. The inclusion of an informant interview is known to enhance the validity of the brief CSID [8]. The CSID informant score was subtracted from the CSID cognitive score to provide the total score (range, − 6 to 9). A combined CSID total score (i.e., after subtraction of the informant score from the cognitive score) ≤4 is also suggestive of possible dementia. Thus, study participants may be classified as screening positive for probable dementia in one of two ways: (a) CSID cognitive score ≤ 4; or (b) CSID cognitive score 5–6 and CSID total score ≤4.

Following screening assessment for dementia, we administered a study questionnaire to elicit other variables, including vascular disease, lifestyle behaviors, psychosocial and psychological factors, infectious disease exposure, and family history. Each variable was enumerated as being either present (yes) or absent (no). Where necessary, a family member provided further clarification during the interview. Study participants’ ages were cross-checked with their national identification cards and/or baptism cards. Formal education was defined as the number of years spent in school: none, 1–7 years (primary school), 8–11 years (ordinary level school), and 12 or more years (advanced level and tertiary education). Family history of dementia was elicited with a single self-report item identifying a first-degree relative (sibling or parents) who had ever showed signs of dementia or received a diagnosis of dementia. Lifetime history of cigarette smoking and alcohol consumption were elicited using self-report. High-fat dietary intake was assessed with a single item eliciting a lifetime history of high-fat intake, defined as regular, frequent consumption of animal products (e.g., milk, meat, ghee) or ground nuts during the ages of 25-45 years. Religious integration was defined as participating in religious activities in addition to community prayer. To assess physical activity prior to 60 years of age, we asked a single question about heavy manual labor, riding a bicycle at least three days a week, or engaging in 30 minutes of exercise at least three times a week. Lifetime history of traumatic head injury was defined as ever experiencing head injury accompanied by loss of consciousness. We also elicited lifetime history of syphilis, tuberculosis, human immunodeficiency virus, bacterial meningitis, cerebral malaria, type II diabetes mellitus, cerebrovascular accident, hypertension, epilepsy, limb paralysis, or thyroid condition. Study participants’ responses were cross-checked against any medical records kept in the study participants’ homes. An adequately ventilated kitchen was defined as one in which the household cooking took place in an open space or one in which there was ventilation from a window or chimney. All study tools were interviewer-administered in the local language (Runyankore-Rukiga). Survey questions were written in English, translated from English into Runyankore-Rukiga, and then back-translated to verify fidelity to the original wording.

### Data management and analysis

Data were entered, cleaned, and analyzed using Epi Info version 7.2.2.6. As described above, the brief CSID provides an estimate of the screening prevalence of dementia. In most settings, the screening prevalence of dementia likely exceeds the true prevalence of dementia, due to the nonzero false positive rate. While the true prevalence of dementia is unknowable in the absence of data from a criterion standard (i.e., clinical diagnosis made on the basis of a structured clinical interview), we followed the “back estimation” method described by de Jager et al. [9] to estimate the prevalence of dementia based on our screening data and the known sensitivity (0.95) and specificity (0.90) of the brief CSID [8]. First, we estimated the number of true positives (TP) given the well-known relationships between sensitivity (SE)/specificity (SP) and the number of test positives (testp) and test negatives (testn):

$$ TP=\frac{testp-\left( testn\times \frac{1- SP}{SP}\right)}{1-\left(\frac{1- SP}{SP}\times \frac{1- SE}{SE}\right)} $$

We then used the estimated number of true positives to calculate the estimated prevalence rate.

We compared the proportions of those with probable dementia across different subgroups using the chi-square test. All variables with a statistically significant association with probable dementia on bivariate analysis were entered as potential covariates in a multivariable logistic regression model specifying probable dementia as the dependent variable.

### Ethics approval and consent to participate

Ethical approval to conduct this study was obtained from the Mbarara University of Science and Technology Research Ethics Committee (reference number 13/10–18). Consistent with national guidelines, clearance for the study was granted by the Uganda National Council for Science and Technology (reference number SS 4842). We obtained administrative permission to enter the study communities from the district administrative head at each site. All study participants (informants and caregivers) provided written informed consent to participate. If a signature could not be obtained for literacy reasons, verbal informed consent was obtained in the presence of a witness, and the study participant was permitted to indicate a signature using a thumbprint. For study participants who could not provide informed consent due to cognitive difficulties (e.g., presumed dementia), informed consent to participate was obtained from a caregiver, and the study participant was permitted to provide assent. All participants who screened positive dementia were referred to the nearest hospital (Mbarara Regional Referral Hospital, Kabale Regional Referral Hospital, Rugarama Hospital [Kabale], Kisiizi Hospital [Rukungiri], Nyakibale Hospital [Rukungiri], Itojo Hospital [Ntungamo], Kitagata General Hospital [Sheema], Kampala International University Teaching Hospital [Ishaka], or Ishaka Adventist Hospital [Ishaka]) for further clinical assessment and management.

## Results

### Characteristics of the study sample

A total of 400 index participants along with 400 caregivers (“informants”) were interviewed. All potential participants approached by our team for interviews agreed to participate. Summary statistics are provided in Table 1 (all percentages may not add up to 100 due to rounding). The mean age of the index participant sample was 72 years (range, 60–108 years). In comparison, the informant/caregiver sample had a mean age of 34 years (range, 13–78 years). Most index participants (238 [60%]) and informants (263 [66%]) were women. Nearly all informants were biologically related to the index participants.

**Table 1:** Socio-demographic characteristics of index participants (*N* = 400) and informants (*N* = 400)

	Variable	Number	Percent
*Index participants (N = 400)*			
Age in years, mean (SD)		72 (9.5)	
Sex	Men	162	41
	Women	238	60
Formal education	None	180	45
	Primary	175	44
	Ordinary level	19	4.8
	Advanced level and above	26	6.5
Marital status	Married	207	52
	Separated/Divorced	29	7.3
	Widowed	155	39
	Never married	9	2.3
Religion	Christian	343	86
	Muslim	57	14
*Informants (N = 400)*			
Relationship to index participant	Spouse	46	12
	Sibling	9	2.3
	Child	144	36
	Grandchild	116	29
	Other relative	62	16
	Not biologically related	23	5.8
Age in years, mean (SD)		34 (15)	
Sex	Men	137	34
	Women	263	66
Years of formal education	None	46	12
	Primary	170	43
	Ordinary level	139	35
	Advanced level and above	44	11
	Not answered	1	0.3

### Prevalence of dementia

Among the 400 index participants, the median CSID cognitive score was 7 (interquartile range [IQR], 6–9), and 23 (5.8%) had a CSID cognitive score ≤ 4. Another 103 (26%) index participants had a CSID cognitive score of 5 or 6. For these 103 participants, a caregiver or close relative was interviewed; the median CSID informant score was 3 (IQR, 2–4), and 83/103 (81%) had a CSID total score ≤ 4. Caregivers most frequently reported that index participants had problems with ‘forgetting where they have put things’ (250 [63%]), ‘general decline in mental functioning’ (223 [56%]), ‘ability to think and reason’ (158 [40%]), and ‘remembering what they did the day before’ (117 [29%]). Caregivers were least likely to report that index participants had ‘difficulties with dressing’ (71 [18%]).

The total number of index participants who screened positive for probable dementia, on the basis of either the CSID cognitive score or the CSID informant score, was 23 + 83 = 106 (27%). Using the back-estimation method described above, the estimated number of true positives was 78, for an estimated prevalence rate of 78/400 = 20%.

### Correlates of probable dementia

Correlates of probable dementia are shown in Table 2. On bivariate analysis, statistically significant correlates of probable dementia included age, education, religious activity, having an adequately ventilated kitchen, and physical activity. When the candidate covariates were entered simultaneously into a multivariable logistic regression model, all retained a statistically significant association with probable dementia except for religious activity. Probable dementia was positively correlated with age (adjusted odds ratio [AOR] = 1.02; 95% confidence interval [CI], 1.01–1.03) and was negatively correlated with education (AOR = 0.68; 95% CI, 0.49–0.96), having an adequately ventilated kitchen (AOR = 0.43; 95% CI, 0.24–0.77), and physical activity (AOR = 0.44; 95% CI, 0.27–0.72).

**Table 2:** Correlates of probable dementia, based on bivariate analysis and multivariable logistic regression

	N	Percent	OR (95%CI)	*P*-Value	AOR (95% CI)	*P*-Value
Sociodemographic factors						
Age (years)	400		0.99 (0.99–0.99)	<0.001	1.02 (1.01–1.03)	<0.001
Male (vs. female)	162	26	0.91 (0.58–1.44)	0.69		
Formal education (yes/no)	220	20	0.43 (0.27–0.67)	< 0.001	0.68 (0.49–0.96)	0.03
Christian (vs. Muslim)	343	28	1.27 (0.67–2.51)	0.44		
Religiously active (yes/no)	183	19	0.45 (0.28–0.71)	<0.001	0.63 (0.38–1.05)	0.08
Household cooking done with firewood (vs. charcoal)	390	27	0.67 (0.14–3.21)	0.89		
Adequate ventilation of kitchen (yes/no)	329	24	0.47 (0.27–0.81)	0.01	0.43 (0.24–0.77)	0.004
Vascular factors						
Hypertension (yes/no)	125	32	1.43 (0.90–2.28)	0.13		
Cerebrovascular accident (yes/no)	17	35	1.50 (0.54–4.17)	0.43		
Diabetes mellitus (yes/no)	38	21	0.71 (0.31–1.59)	0.40		
Behavioral factors						
High-fat diet during middle age (yes/no)	209	28	1.08 (0.70–1.69)	0.72		
Physically active (yes/no)	246	19	0.34 (0.22–0.54)	<0.001	0.44 (0.27–0.72)	0.001
Cigarette smoking (yes/no)	183	27	1.03 (0.66–1.60)	0.89		
Alcohol use (yes/no)	233	26	0.90 (0.57–1.40)	0.64		
Infectious factors						
Human immunodeficiency virus (yes/no)	7	43	2.06 (0.45–9.35)	0.60*		
Meningitis (yes/no)	12	33	1.38 (0.41–4.68)	0.60		
Cerebral malaria (yes/no)	28	32	1.31 (0.57–2.98)	0.53		
Syphilis (yes/no)	66	24	0.84 (0.46–1.55)	0.58		
Tuberculosis (yes/no)	15	40	1.85 (0.64–5.33)	0.25		
Other factors						
Traumatic head injury (yes/no)	27	30	1.15 (0.49–2.71)	0.75		
Limb paralysis (yes/no)	204	26	0.90 (0.58–1.40)	0.64		
Epilepsy (yes/no)	9	56	3.50 (0.93–13.3)	0.12*		
Thyroid condition (yes/no)	14	21	0.73 (0.20–2.67)	0.86		
Family history of dementia (yes/no)	80	20	0.62 (0.34–1.13)	0.11		

*Yates *P* value

## Discussion

In this population-based study of 400 older-age adults from rural Uganda, we estimated the population prevalence of dementia to be 20%. This estimated prevalence is higher than the 8% estimated prevalence rate reported in a recently published community-based survey from South Africa, which had a similar age distribution of study participants and used a similar screening instrument (i.e., the brief CSID) [9]. Notably, in our study, nearly 80% of positive dementia screens were categorized as such due to the informant/caregiver score, which exceeds the 30% reported by de Jager et al. [9]. The difference in prevalence rates between our study and de Jager’s study is therefore due to differences in the distribution of informant scores. Assessment tools which incorporate an informant interview, like the CSID, can be affected by the reliability of the informant. In our study, informants provided relatively high scores. In addition, the involvement of village health team members in mobilizing study participants could have led informants to anticipate aid from study investigators and thereby provide exaggerated responses.

Our estimate of dementia prevalence also exceeds the rate of 6.4% obtained by Longdon et al. in Tanzania, which had an older age distribution but which also used the full CSID and *Diagnostic and* *Statistical Manual of Mental Disorders* (fourth edition) criteria to obtain clinical diagnoses [10]. Thus, the observed discrepancy between the Longdon et al. study and ours is to be expected, given that screening instruments generally yield high prevalence estimates compared with structured clinical interviews (which is generally the case for depression [11, 12]). In addition, as argued by Paddick et al., the CSID screening process generally produces higher prevalence estimates compared with structured clinical interviews possibly due to the detection of early dementia and mild cognitive impairment [13].

Our study identified several independent correlates of probable dementia. As expected, advancing age had a statistically significant positive correlation with probable dementia, similar to what has been demonstrated in other studies [9, 10, 14]. Also consistent with prior work, formal education was inversely associated with probable dementia [13–15], although this association has not been universally observed [9, 10, 16]. Although there were high levels of illiteracy in our study and thede Jager et al. study, formal education was inversely associated with probable dementia in our study whereas it did not have the same association in the de Jager study [9]. This difference could be due to differences in informal lifestyle choices shaped by culture that are thought to be protective against cognitive decline.

Physical activity, which we found to be inversely associated with probable dementia, has not been widely studied in relation to brain health in sub-Saharan Africa. Finally, we also found that having an adequately ventilated kitchen had an independent, inverse association with probable dementia. Air pollution is a growing problem globally [17] and has been linked, in studies from Uganda, to other health problems like respiratory symptoms [18, 19]. One population-based study from Taiwan found that higher levels of carbon monoxide exposure were significantly associated with an increased risk of dementia [20]. People in rural Uganda may be exposed to indoor air pollution when they sit around the fire for warmth during the evenings or when they prepare their meals in inadequately ventilated kitchens. Prior work by Saenz et al. has identified an inverse association between indoor air pollution and cognitive function [21].

Interpretation of our findings is subject to several limitations. First, due to limited resources, we could not obtain clinical diagnoses of dementia using structured clinical interviews. Because we used the brief CSID, we may have overestimated the prevalence of dementia, given that screening instruments generally produce higher prevalence estimates due to the detection of early dementia and mild cognitive impairment. However, the brief CSID has shown strong evidence of validity in screening for dementia, and we used the back-estimation method described by de Jager et al. [9] which revised downward the estimated prevalence. A second limitation is that early and midlife exposure variables were measured with self-report. Cognitive impairment could have caused study participants to recall these variables with error.

These limitations notwithstanding, understanding the prevalence and correlates of AD/RD in a resource-constrained setting like Uganda is critical for creating public awareness, influencing policy recommendations, and developing effective interventions.

## Conclusions

In this population-based sample of older-age adults, nearly one-fifth screened positive for dementia. We found a relatively high prevalence of dementia compared with studies conducted elsewhere in sub-Saharan Africa. Further studies are needed to establish the reasons underlying this discrepancy, which could have resulted from artefact, or from better case finding, longer survival of persons with dementia, and/or regional differences in the epidemiology of dementia and cognitive impairment. In addition, further studies to determine the primary causes and subtypes of dementia need to be done to inform specific risk-focused interventions.

## Data Availability

The full dataset generated and analyzed during the current study are not publicly available in order to maintain the privacy of the individuals interviewed during this study. De-identified data can be made available by the corresponding author on reasonable request.

## Abbreviations

AD/RD: Alzheimer’s disease and related dementias
AOR: Adjusted odds ratio
CI: Confidence interval
CSID: Community Screening Instrument for Dementia
IQR: Interquartile range
